# *QuickStats:* Percentage of Suicides* and Homicides^†^ Involving a Firearm Among Persons Aged ≥10 Years, by Age Group — United States, 2022

**DOI:** 10.15585/mmwr.mm7337a3

**Published:** 2024-09-19

**Figure:**
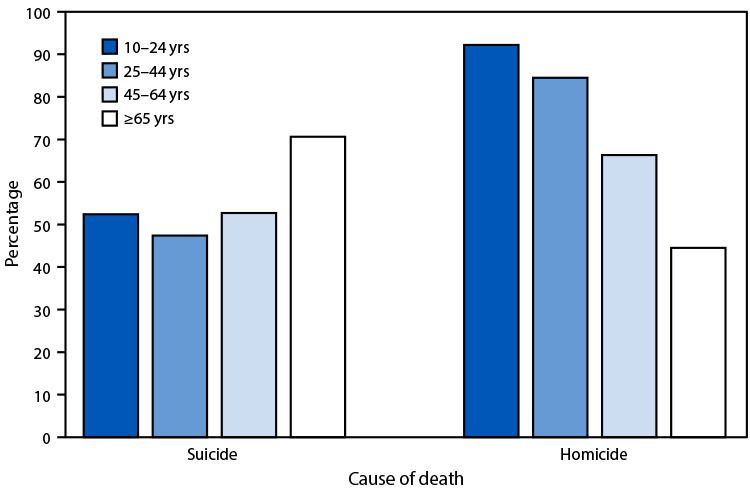
In 2022, among persons aged ≥10 years, the percentage of suicide deaths involving a firearm was lowest among persons aged 25–44 years (47.4%) and highest among persons aged ≥65 years (70.6%). The percentage of homicide deaths that involved a firearm was highest among persons aged 10–24 years and then decreased with age, from 92.2% among those aged 10–24 years to 44.5% among those aged ≥65 years.

**Supplementary Table:** https://stacks.cdc.gov/view/cdc/160513

**Source:** National Center for Health Statistics, National Vital Statistics System, Mortality Data, 2022. https://www.cdc.gov/nchs/nvss/deaths.htm

For more information on this topic, CDC recommends the following links: https://www.cdc.gov/suicide and https://www.cdc.gov/firearm-violence/about/index.html.

